# Diel timing of nest predation changes across breeding season in a subtropical shorebird

**DOI:** 10.1002/ece3.8025

**Published:** 2021-09-08

**Authors:** Martin Sládeček, Kateřina Brynychová, Esmat Elhassan, Miroslav E. Šálek, Veronika Janatová, Eva Vozabulová, Petr Chajma, Veronika Firlová, Lucie Pešková, Aisha Almuhery, Martin Bulla

**Affiliations:** ^1^ Faculty of Environmental Sciences Czech University of Life Sciences Prague Prague Czech Republic; ^2^ Natural Resources Conservation Section Environment Department Dubai Municipality Abu Hail, Dubai United Arab Emirates; ^3^ Department of Behavioural Ecology and Evolutionary Genetics Max Planck Institute for Ornithology Seewiesen Germany

**Keywords:** continuous monitoring, diel pattern, diel timing, nest predation, predation rate, red‐wattled lapwing, shorebirds, survival analyses, timing of predation, waders

## Abstract

Predation is the most common cause of nest failure in birds. While nest predation is relatively well studied in general, our knowledge is unevenly distributed across the globe and taxa, with, for example, limited information on shorebirds breeding in subtropics. Importantly, we know fairly little about the timing of predation within a day. Here, we followed 444 nests of the red‐wattled lapwing (*Vanellus indicus*), a ground‐nesting shorebird, for a sum of 7,828 days to estimate a nest predation rate, and continuously monitored 230 of these nests for a sum of 2,779 days to reveal how the timing of predation changes over the day and season in a subtropical desert. We found that 312 nests (70%) hatched, 76 nests (17%) were predated, 23 (5%) failed for other reasons, and 33 (7%) had an unknown fate. Daily predation rate was 0.95% (95%CrI: 0.76% – 1.19%), which for a 30‐day long incubation period translates into ~25% (20% – 30%) chance of nest being predated. Such a predation rate is low compared to most other avian species. Predation events (*N* = 25) were evenly distributed across day and night, with a tendency for increased predation around sunrise, and evenly distributed also across the season, although night predation was more common later in the season, perhaps because predators reduce their activity during daylight to avoid extreme heat. Indeed, nests were never predated when midday ground temperatures exceeded 45℃. Whether the diel activity pattern of resident predators undeniably changes across the breeding season and whether the described predation patterns hold for other populations, species, and geographical regions await future investigations.

## Graphical abstract

The diel timing of nest predation in birds is mostly unknown. Here, incubating red‐wattled lapwings faced a low chance of nest predation (20%–30%), but night predation was more common later in the season, perhaps because predators reduce their activity during daylight to avoid extreme heat. Indeed, nests were never predated when midday ground temperatures exceeded 45℃. These results highlight the need for continuous monitoring to reveal the temporal pattern of predation on multiple time scales.

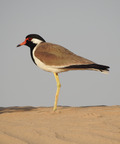

## 1 INTRODUCTION

Predation affects the reproduction of wild populations (Caro, 2005; Ricklefs, 1969; Skutch, 1985). Indeed, predation is the most common cause of nest failure in birds (Ricklefs, 1969; Skutch, 1985). While nest predation is relatively well studied in general, our knowledge is biased toward the Northern hemisphere temperate and arctic regions (Bulla et al., 2019; Freeman et al., 2020; Kubelka, Šálek, et al., 2018; Unzeta et al., 2020), which hampers global comparative analyses. Moreover, regardless of the region, we know fairly little about when within a day nests are predated (Tulp et al., 2001; hereafter “diel timing of nest predation”; Praus & Weidinger, 2010; Weidinger, 2010; DeGregorio et al., 2015; Brynychová et al., 2020; Laidlaw et al., 2020), perhaps because it requires continuous nest monitoring (Pietz et al., 2012; Weidinger, 2006).

Knowing when nests of a given species or population are predated may help in interpreting various behaviors of incubating parents, such as the timing of breeding season (Morton, 1971), pattern of nest attendance (Bakner et al., 2019; Cervencl et al., 2011; Kasun B Ekanayake et al., 2015; Massaro et al., 2008; Skórka et al., 2012; Sládeček et al., 2019), or daily rhythms of self‐maintenance activities (Brynychová et al., 2020; Javůrková et al., 2011). Notably, given the lack of information on diel timing of nest predation, it is unclear whether there is a population‐ or species‐specific, latitudinal, or habitat‐dependent pattern in the timing of predation. For example, is there a day–night nest predation pattern around the equator and around the clock nest predation toward the poles, where it is light 24 hr a day during the breeding season?

Diel timing of nest predation for a given avian species likely depends on its anti‐predatory strategy (Brynychová et al., 2020; Bulla et al., 2016; Eggers et al., 2008), as well as on when its main predator species are active (DeGregorio et al., 2015; Kämmerle et al., 2020). For example, corvids *(Corvidae)* are active and search for their prey during daylight hours (Tahajjul Taufique et al., 2016), but ground‐nesting northern lapwings *(Vanellus vanellus)* actively protect their nests by chasing away corvids (and other daylight active avian predators). Thus, nests of northern lapwings are rarely predated during the day, and night predation prevails (Brynychová et al., 2020). In contrast, temperate open‐cup nesting and ground‐nesting passerines do not actively defend their nests and consequently, both mammals and birds predate their nests, resulting in around the clock nest predation (Praus & Weidinger, 2010; Weidinger, 2010). In general, mammalian predators are nocturnal and predate nests and incubating parents at night, while avian nest predators are active during daylight and are the main daylight predators (Weidinger, 2010). In contrast, snakes, which are common predators of avian nests in the tropics (Robinson et al., 2005; Visco & Sherry, 2015) and parts of temperate North America (Weatherhead & Blouin‐demers, 2004), predate nests around the clock (DeGregorio et al., 2015). Importantly, the frequency of nest predation may also change over the breeding season. Such change may coincide with changes in vegetation density and nest concealment (Batáry et al., 2004; Mezquida & Marone, 2001; Morton, 1971; Sieving, 2019), and with changes in the presence of main predators, for example, due to migration or due to dispersal of new generations (Patnode & White, 1992; Sloan et al., 1998; Sperry et al., 2008).

Here, we estimated nest predation rate and investigated temporal dynamics of nest predation in the red‐wattled lapwing *(Vanellus indicus)*, a shorebird breeding in an arid and hot subtropical environment. Specifically, we followed 444 nests south of Dubai, United Arab Emirates, for a sum of 7,828 days to estimate daily and total nest predation rate, as well as change in daily predation rate across the breeding season. We also continuously monitored 230 of these nests for a total of 2,779 days to reveal the diel timing of nest predation and its changes over the breeding season.

**Figure 1:**
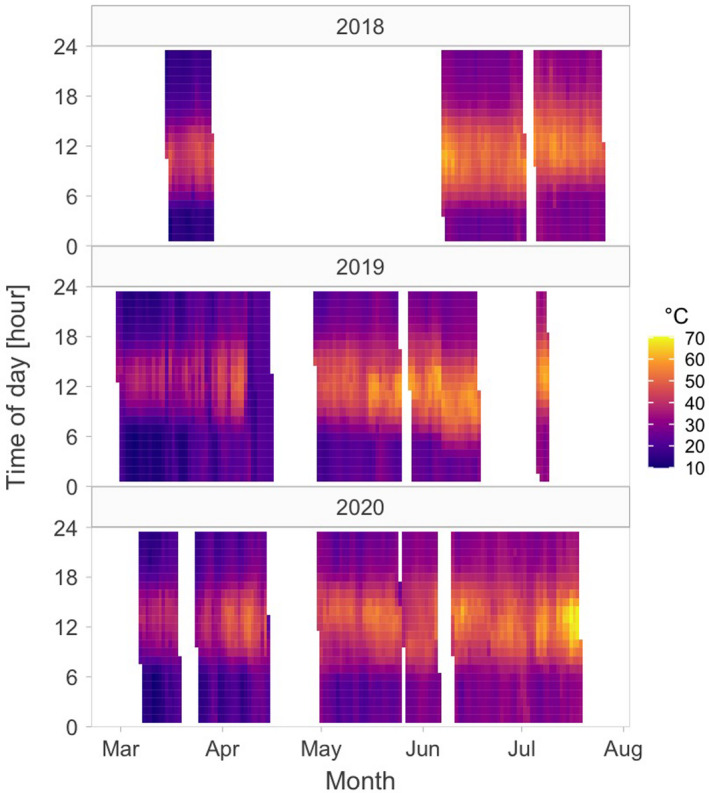
Changes in hourly ground temperatures across day and season. Depicted are median hourly ground temperatures in the study area based on all recordings of sensors located next to the nests at a given hour (see Methods for details). White space indicates no temperature recordings

We tested the following three predictions. First, we expected daytime nest predation to be less common than night‐time nest predation because red‐wattled lapwings actively defend their nests during the day (but not during the night) by alarm calling when a predator is at a great distance and by attacking a predator, often in cooperation with nearby breeding pairs (Kaur & Khera, 2017; Narwade et al., 2010). Second, we expected nest predation to decrease over the season because some overwintering avian predators migrate out and migrating avian predators pass through the study area early in the lapwing's breeding season (eBird, 2020) and because other avian chicks—an alternative prey to red‐wattled lapwing nests—are available later in the breeding season (personal observation). Third, we expected daylight nest predation (if any) to decline over the breeding season, because the presence of migrating avian predators—daylight predators of nests—declines over the breeding season (Table A1 eBird, 2020) and because ambient and ground temperatures increase dramatically over the breeding season (Figure 1) to the point where midday activity of most endotherm animals is close to impossible (Abdu et al., 2018; Albright et al., 2017; Streicher et al., 2017).

## 2 METHODS

### 2.1 Study site and species

**Figure 2:**
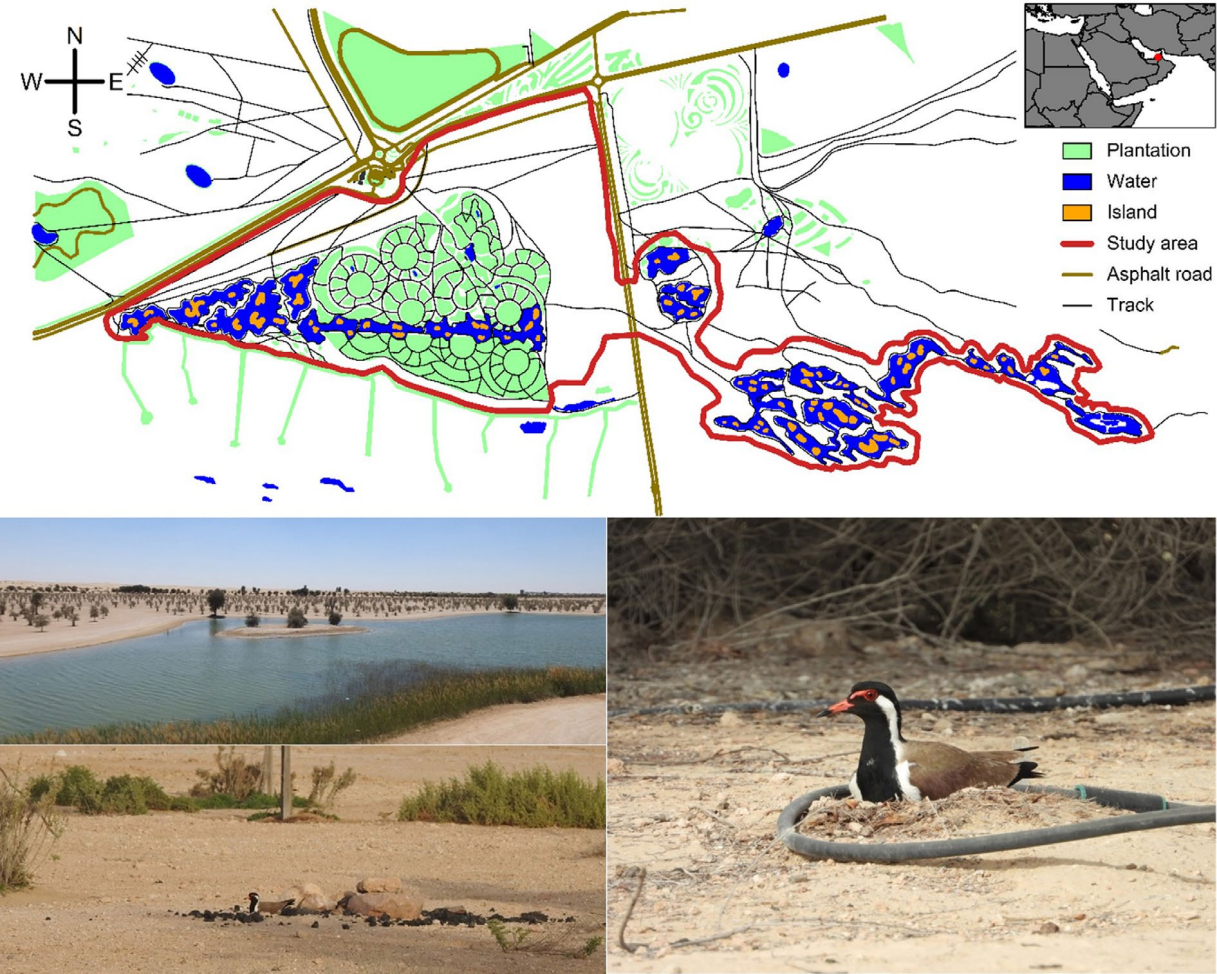
Study site and illustration of how readily visible are incubating red‐wattled lapwings. Note that the lapwings often breed close to irrigation pipes (right picture). Map based on ©OpenStreetMap contributors and our digitalization of the study site. Pictures: ©Miroslav E. Šálek

The study was conducted in the central part of Al Marmoom Conservation Reserve, Dubai, United Arab Emirates (24.84°, 55.36°), during the 2018 – 2020 breeding seasons. The reserve hosts broad and rich array of animal communities, including nest predators (Table A1). The 6.6km^2^ study area is in the heart of the reserve and consists of 26 artificial lakes, artificial plantations of desert shrubs and trees, and dunes (Figure 2).

The red‐wattled lapwing is a poorly studied ground‐nesting shorebird species that breeds mainly in human‐altered habitats such as corn and grass fields, larger gardens, or waste, fallow and plouwed land (Wiersma, 2020). Their global population is stable (not endangered, Wiersma, 2020) and growing on the Arabian Peninsula (Symes et al., 2017). The local red‐wattled lapwing population consists of approximately 80 breeding pairs. The breeding season lasts from early February to the beginning of August, and some individuals have several (up to 5) breeding attempts (our unpublished data). The red‐wattled lapwings nest on islands and within the tree plantations in the vicinity of lakes. They build their nests on the ground. Incubating parents are readily visible on the nest from afar (Figure 2). Both parents continuously attend the nest and nests are rarely left unattended. When eggs hatch, parents remove large eggshells and take those far away from the nest. Precocial chicks leave the nest shortly after hatching (Wiersma, 2020, our observation). Families with chicks remain in the vicinity of the nest until fledging. We never observed chicks further than ~300 m from the nest and, with one exception, never on a different island than the one, on which they hatched.

### 2.2 Nest monitoring

We searched for nests by slowly driving a car through the study area, looking for incubating adults that are readily visible from a distance (Figure 2). We used this same noninvasive method to monitor nests during incubation. One observer (Esmat Elhassan) searched for nests and checked nests at least once (but usually 2–3 times) a week, across the whole breeding seasons. The rest of the research team searched for nests daily during two‐ to six‐week‐long expeditions (1–3 expeditions per year). Given the frequency of our visits and the visibility of incubating parents, we likely found most lapwing nests within the study area and followed most of the parents that guided their chicks. Upon finding a nest, we measured and floated the eggs to estimate when a clutch was initiated and likely to hatch (see below).

We trapped adults on nests using spring traps triggered from a distance by a fishing line and marked the adults with a unique combination of metal and 4 color rings and a green flag embedded with a glass passive integrated transponder (Biomark: Ø 2.1 × 9.0 mm, 0.087 g, ISO FDXB, http://www.biomark.com/, see Appendix Picture A1 in (Bulla et al. 2013); or Smartrac 704487–09 Glass tag Ø 2.12 × 12 mm, https://rfid.averydennison.com). The transponder enabled us to determine the presence of the specific bird on the nest. We took a small (ca. 50 μl) blood sample from a brachial vein for sexing and left the nest undisturbed for at least one day between consecutive catching attempts. We then attempted to visit the nests at least once a week and around the estimated hatch date to monitor and determine their fate. Possible nest fates were hatched (at least one egg hatched), predated (includes also partial predation events after which parents abandoned their nest), abandoned, or failed for other reasons (e.g., trampled or dead embryos due to overheating), and unknown.

We continuously followed (at least for some time) 230 nests with one incubation monitoring system or with a combination of incubation monitoring systems: 35 nests were monitored with a video recording system (Sládeček et al., 2019), 188 nests with data loggers that recorded temperature and humidity inside and outside of the nest in 1‐s intervals (DHT, http://berg.fzp.czu.cz) or recorded only temperature in 1‐min intervals (Tinytag Talk 2, Bulla et al., 2014), 144 nests were monitored with a radio frequency identification device (RFID) that detected a passive transponder of an incubating parent in 5‐s interval (Bulla et al., 2014), 40 nests with multisensory data logger that recorded temperature and humidity inside and outside of the nest and also detected passive transponders in 1‐s intervals (ZAYDA 1.1, http://berg.fzp.czu.cz), and 15 nests with a dummy egg recording temperature and acceleration in 1‐s to 30‐s intervals (ANITRA, https://anitracking.com). The dummy eggs were placed into the nests with less than 4 eggs and were accompanied by a temperature logger (DHT 2.1) placed in the vicinity of the nest. The temperature‐humidity data loggers (DHT 2.1) and multisensory loggers (ZAYDA 1.1) were installed similarly to the other temperature loggers and RFIDs (Picture A1 and Bulla et al., 2014).

We visualized and inspected all recordings to identify the data with device‐caused errors and periods when a bird had removed a sensor from the nest, that is, when a sensor had recorded outside, not inside nest parameters.

### 2.3 Clutch initiation and fate

We defined “clutch initiation” as the day when the first egg was laid, which also indicates the onset (day one) of the incubation period because red‐wattled lapwings incubate their eggs and protect them against the extreme heat as soon as the first egg is laid (own observation). We assumed (based on our observations) that females lay eggs in 1.5‐day intervals and hence that females finish laying a 4‐egg clutch in 4.5 days. We further assumed a 30‐day long incubation period from “clutch initiation” until the first egg hatches (mean = 30, median = 31, range: 25–34; *N* = 13 hatched nests found at laying). Thus, if a nest was found during egg‐laying (*N* = 80) we estimated “clutch initiation” by subtracting the number of days it took to lay the clutch (e.g., for 3 eggs, 3 days; 1.5*(found clutch size−1)) from the date the nest was found. If a nest was found with a complete clutch, we estimated “clutch initiation” as the date when the oldest egg was laid based on the floating of the eggs (van Paassen et al., 1984). We calculated the “estimated hatch date” as “clutch initiation” plus 30 days.

We considered nests as hatched (*N* = 312), when at least one chick hatched, based on observations of (i) at least one chick on or around the nest during the final nest‐check (*N* = 197 nests), (ii) color‐marked parents guiding chicks after the final nest visit (*N* = 36 nests), or (iii) small (≤ 5mm) eggshell pieces in the nest that result from a chick chipping its way out of its egg (*N* = 79; Brown et al., 2014; Mabee et al., 2006). The use of eggshell pieces has been used to define successfully hatched nests in other shorebird species (Kentie et al., 2015; Laidlaw et al., 2020). Importantly, we ringed 233 chicks whose identity was unknown as they were found away from nests. Most of these 233 chicks likely came from the 79 nests where we assumed hatching based on eggshell pieces, because (a) we follow nearly all nests within the study area, (b) families with chicks stay within the study area as chicks would die in the surrounding desert, and (c), with one exception, we have never observed chicks from a known nest on an island other than the one they hatched at. Convincingly, when we assume that the 233 chicks with an unknown nest identity came from the 79 nests where hatching was determined from eggshell pieces, the average number of chicks per nest is 2.95, which closely corresponds with an average of 2.75 chicks per nest in nests with known chick identity (641 chicks from 233 nests). Moreover, these 79 nests where we identified hatching based on tiny eggshell pieces have little influence on our estimation of predation rate; treating these nests as unknown (i.e., observation period ending at the last visit when the nest was still active) generates similar predation rate as when we treat these nests as hatched.

We estimated the hatch date in the following way and order. First, we assumed that the nest hatched 1 day ago if we knew when the chicks left the nest (a) based on the visualized continuously recorded data (*N* = 91) or (b) freshly hatched chicks found around the nest (*N* = 24). Second, we assumed that the nest hatched 12 hr earlier than our nest visit if during the nest visit both eggs and chicks were found in the nest (i.e., eggs were in the process of hatching; *N* = 50). Third, if a nest was found empty but with signs of hatching (*N* = 57), older chicks were found around the nest during the final nest visit (*N* = 73), or parents were found later with chicks (*N* = 14), we assumed that nest hatched on the estimated hatch date (*N* = 144), unless the estimated hatch date was earlier than the last visit when the nest was seen active (without signs of hatching), in which case we assumed that chicks hatched one day after such visit (*N* = 3). Finally, 18 nests were discovered during or shortly after hatching (chicks in the nest cup). These 18 nests were not used in the analyses.

**Figure A1:**
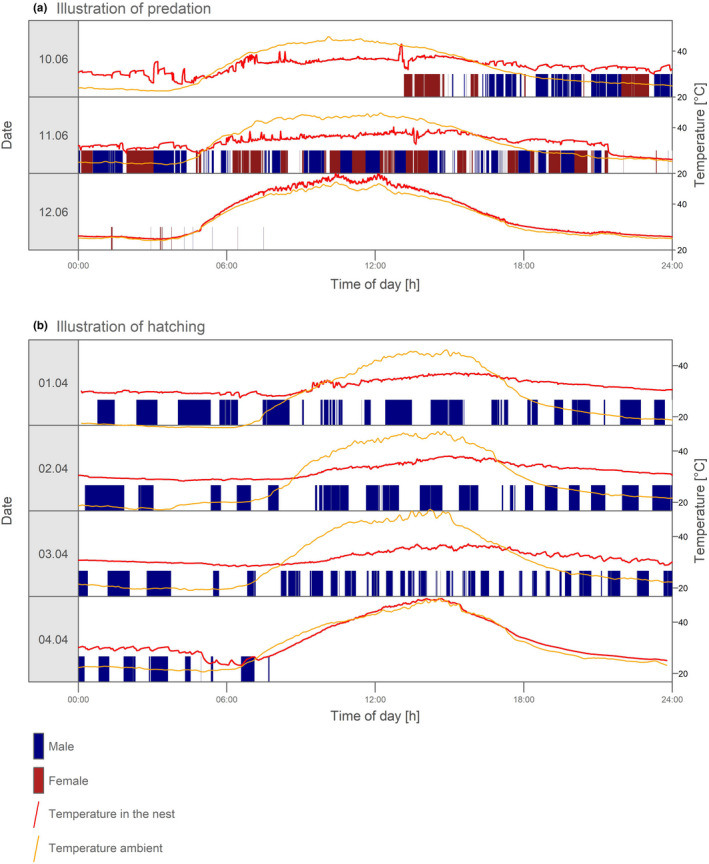
Illustration of predation and hatching on continuous recordings from red‐wattled lapwing nests. (a, b) Red line indicates nest temperatures, orange line ground temperatures next to the nest. Bars indicate RFID recording of female (dark red) and male (blue) on the nest. (a) Nest was predated in the evening of June 11 when nest temperatures quickly drop and reach ground temperatures. Despite the nest predation, parents still occasionally passed over the nest until the morning of June 12. (b) Nest hatched in the morning on April 4. Note the difference between the changing nest temperatures in the morning on April 4 (indicating chick presence on the nest) and smooth nest temperatures in the morning on April 3. The change in temperature and RFID pattern is visible even if only a single parent (here male) is tagged

We considered nests as predated (*N* = 76) when nests were found (a) empty without signs of hatching, that is, without tiny eggshell pieces that indicate hatching, and if parents were ringed, they were never seen with chicks and did not alarm call (*N* = 69; for 25 of these nests predation was also confirmed by the continuous recording, which indicated the abrupt end of incubation, as described below and visible in Figure A1), when nests were found (b) with remains of predated eggs (*N* = 5) or (c) with some eggs missing and some eggs abandoned (no parents around) before expected hatching (i.e., partially predated *N* = 2). Note that red‐wattled lapwings continuously incubate or shade the nest to prevent overheating of the embryos; hence, abandoned (unattended) nest are obvious. Moreover, incubating parents arrange the pyriform eggs with sharp ends to the middle of the nest (Picture A1b). Thus, whenever we were suspicious of nest abandonment, we turned the eggs with the sharp ends out. If during the next visit the eggs remained the way we have left them, the nest was surely abandoned.

For the nests that were not continuously monitored, we estimated the date of predation as a midpoint between the last time when the nest was seen alive and the last nest visit, that is, visit when the nest fate was determined, unless the expected hatch date was earlier, in which case the date of predation corresponds to the expected hatch date (*N* = 43). If the last time when the nest was seen alive was after the expected date of hatching, we assumed that the nest was predated one day after such visit (*N* = 8).

For the continuously monitored predated nests (*N* = 25, none of which was detected by video camera), we estimated the date and time of predation as the time when incubation temperature and humidity abruptly changed and reached the temperature and humidity values recorded outside of nests or as the time when the incubating parent was last recorded with the RFID (Figure A1a). We considered this as the time of predation even if one of the parents visited the nest shortly after the predation event (Figure A1a). The nest fate estimated from temperature loggers matches well with the nest fate recorded by cameras (Weidinger, 2006). In addition, although the RFID method is less precise when only a single parent is tagged with a passive transponder and the nest temperature is not recorded (*N* = 3 nests), the parents exchange frequently on the nest (~hourly; Figure A1), so the bias in the estimated time of predation should be minimal.

We define an observation period as a number of days for which a nest was followed and survived. Thus, the observation period starts when we found the nest and ends with the estimated date of hatching or predation. For nests that failed for other reason than predation (e.g., with infertile eggs or trampled; *N* = 23) or nests with unknown fate (e.g., covered by sand after a windy day; *N* = 33), the end of the observation period indicates the last time when a nest was seen alive (based on visits or logger data). This approach resulted in 39 additional nests with no observation period (15 failed for other reason and 24 with unknown fate); these 39 nests were not used in the analyses.

### 2.4 Ground temperatures

To investigate the relationship between the timing of predation and ambient temperature, the temperature loggers used for continuous nest monitoring recorded also ground temperatures next to the nest. We used these data to compute hourly median, mean, min, and max temperature for each hour and each day of the year, for which we had the data (Figure 1). For the 25 predated nests, we then assigned a median ground temperature during the hour when the nest was predated, as well as a median midday ground temperature of the day when the nest was predated.

### 2.5 Data analysis

#### 2.5.1 General procedures

All statistical analyses and visualizations were performed in R 4.0.2 (R‐Core‐Team, 2019). The figures were created with the “ggplot” function from the “ggplot2” R package (Wickham, 2016). Whenever we fitted linear and generalized models, we used the “sim” function from the “arm” R package and noninformative prior distribution (Gelman & Hill, 2007; Gelman et al., 2016) to create a sample of 5,000 simulated values for each model parameter (posterior distribution). We then reported the effect sizes and model predictions by the medians and the uncertainty of the estimates and predictions by Bayesian 95% credible intervals (95%CrI) represented by 2.5 and 97.5 percentiles of the posterior distribution of the 5,000 simulated or predicted values.

#### 2.5.2 Daily nest predation rate

We estimated the daily nest predation rate according to Mayfield (1961) using “logistic regression” with a number of days in which a nest was predated (0 or 1) and a number of days in which a nest survived as a binomial denominator (Aebischer, 1999). We then calculated a total nest predation rate (a chance of a nest being predated over the whole incubation period) as 1‐(1‐daily predation rate)^30 days‐long incubation period^ (Mayfield, 1961). We further tested whether the daily predation rate changed over the breeding season (“day of the year”). “Day of the year” reflects the midpoint of the period for which each nest was observed. We then compared the fit of the two models by Akaike's Information Criterion corrected for sample size (Anderson, 2008) generated by the “AICc” function from the “MuMIn” R package (Bartoń, 2019).

Of the 192 banded individuals, some were recorded at multiple nests: 35% (67 adults) at two nests, 14% (27 adults) at three nests, 5% (11 adults) at four nests, and 8% (16 adults) at >4 nests. In an attempt to control for this nonindependence of data points, we refitted the models and included female, male, and pair identities as random intercepts, while treating birds at nests with unringed parents as unique identities. Such models did not converge or provided nonsensical estimates. The same was the case for models with only pair identity, only male identity, or only female identity. Our simulations revealed that this was due to the low number of nests associated with a particular pair or bird (i.e., low number of multiple observations per random factor level). Consequently, we used models without random intercepts and acknowledge that some nests may not be independent of each other—an issue common to most studies of daily nest predation (McGuire et al., 2020; Meyer et al., 2020; Weiser et al., 2016, 2018). Importantly, this issue seems negligible because when we removed the known re‐nesting events within each year (i.e., used only the first nesting attempts, where known), the estimate and uncertainty in daily and total predation rate were similar to those from our original model (see Results).

#### 2.5.3 Diel timing of predation

**Figure 3:**
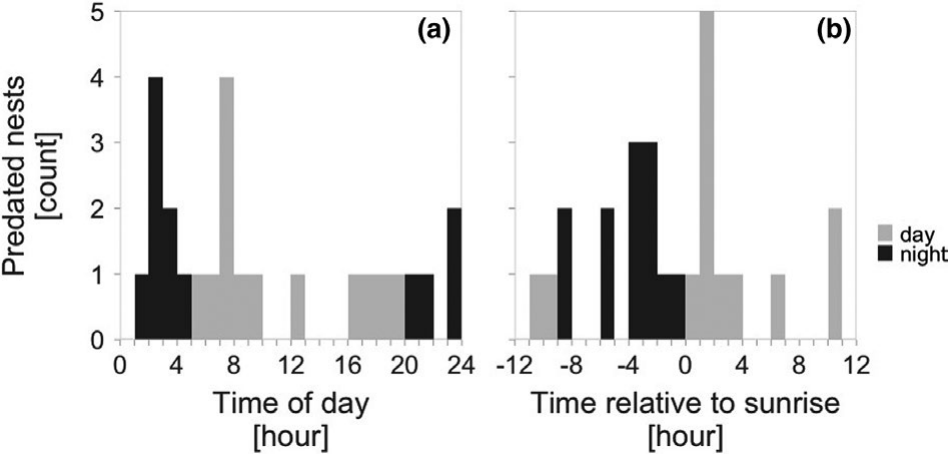
Diel timing of nest predation on red‐wattled lapwing nests. Distribution of nest predation events across the day (a) and in relation to sunrise (b) with night predation (sun >6° below the horizon) in black, daylight predation in gray. Each bar represents a single hour

For the predation events with known timing (*N* = 25 cases), we visualized their distribution across the day, season, and temperature. We then used a generalized linear model with a Poisson error distribution to test whether the number of predation events (count per hour) changed over the day. To account for circular properties of time, time (in hours) was transformed to radians (2 × time × π/2) and fitted as sine and cosine of radians (Bulla et al., 2016). Since the hourly distribution of predation events was centered around sunrise (Figure 3), we also tested whether the probability of predation increased around sunrise by specifying time relative to sunrise (absolute hours) as a continuous predictor.

To further investigate whether the distribution of night and day predation changed over the season, we classified a predation event as “night” when the sun was at least 6° below the horizon (which demarcates the end of the “civil twilight” in the evening and its start in the morning), else we classified the predation event as “day.” The start and the end of each night were estimated for the latitude and longitude of the study site with the “crepuscule” function from the “maptools” R package (Bivand & Lewin‐Koh, 2020). We then fitted a binomial generalized linear model to test how the probability of night predation—a binary response with 1 (night) and 0 (day)—changed over the “breeding season” (day of the year when predation occurred).

The temperatures at the study site increase dramatically over the breeding season (Figure 1; midday temperatures strongly positively correlated with the breeding season: r_Pearson_ = 0.87). To investigate whether the probability of night predation changed with increasing midday temperatures, we explored the relative importance of breeding season and midday temperatures by specifying and comparing three alternative models. First, within the same model we fitted breeding season and midday temperature as predictors. Second, to evaluate whether the effect of breeding season (based on the first model) is confounded by midday temperature, we specified a model with breeding season and residual midday temperature as predictors. The residual midday temperature represents residuals of a model with the midday temperature as a response and the breeding season as a predictor. Third, to evaluate whether the effect of midday temperature (based on the first model) is confounded by breeding season, we specified a complementary model with the midday temperature and the residual breeding season as predictors. The residual breeding season represents residuals from a model with the breeding season as a response and the midday temperature as a predictor. Apart from comparing the change in the effect sizes, we have also compared the model fits by Akaike's Information Criterion corrected for sample size (Anderson, 2008).

As Gaussian models are robust against the violation of model assumptions and perform well on data with the binomial and Poisson distributions (Knief & Forstmeier, 2018; Schielzeth et al., 2020), we also refitted the models on the diel pattern of predation (Table A3) and models on the probability of night predation (Table A4) with Gaussian error distribution. Such models generated similar results to the initial generalized linear models (Table A3, A4).

## 3 RESULTS

During 2018–2020, we followed 444 nests, of which 76 nests (17%) were predated, 312 (70%) hatched, 23 (5%) failed for other reason, and 33 (7%) had an unknown fate. We followed the 444 nests in total for 7,828 days (median = 17.4, mean = 17.6, range: 0.3–67.8 days per nest). Note that some parents truly incubated (likely infertile eggs) for excessively long periods. The daily nest predation rate was 0.95% (95%CrI: 0.76%–1.19%, Table A2), which for a 30‐day long incubation period translates into ~25% (20%–30%) chance of nest being predated. The estimation of daily nest predation rate was insensitive to the exceptionally long observation periods, nests with unknown fate, or multiple nesting attempts. In other words, when we limited the long observation periods (in 45 nests) to 30 days, predation rate was 0.99% (0.78%–1.24%), when we removed the 33 nests with unknown fate, predation rate was 1.02% (0.81%–1.29%), and when we used only the first nesting attempts, where known, predation rate was 0.93% (0.74%–1.17%, *N* = 456 nests). The probability of nest predation did not change over the breeding season, and the model with breeding season was three times less likely than the simple model without breeding season, that is, the simple model fitted the data better (Table A2).

**Figure 4:**
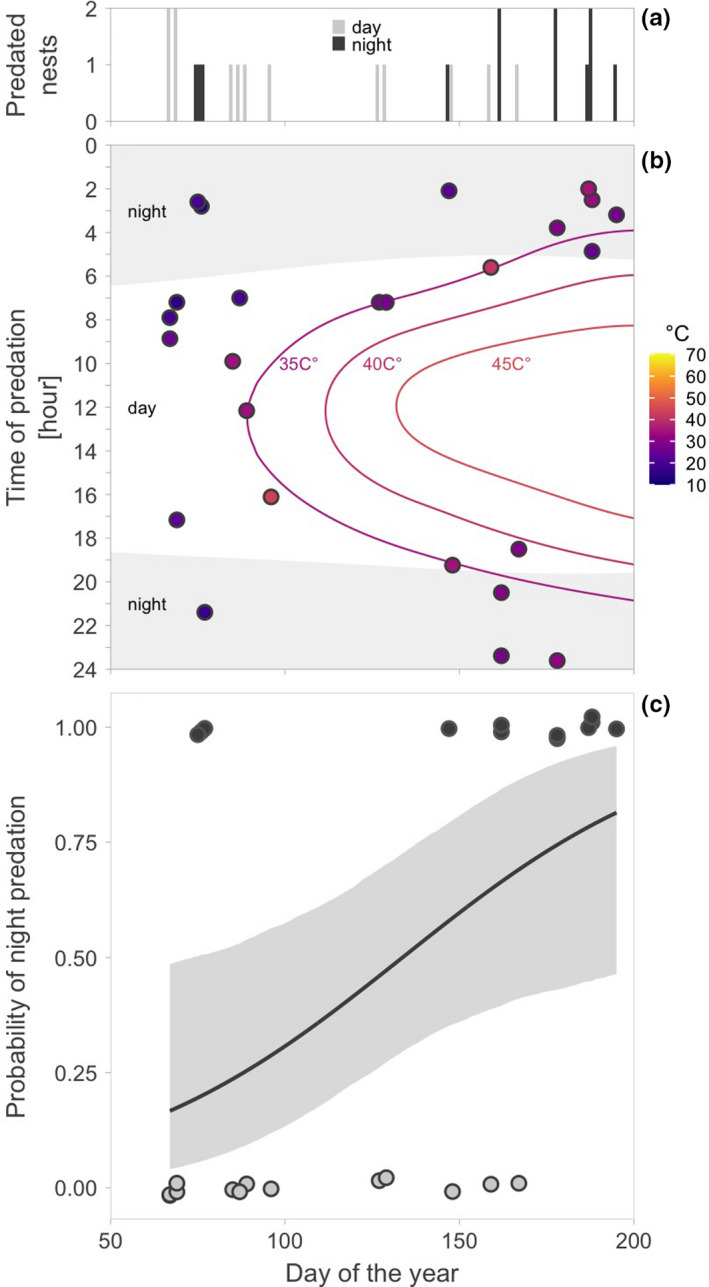
Change in diel timing of predation on red‐wattled lapwing nests across the breeding season. Distribution of nest predation events across the breeding season (day of the year), gray color indicating the day and black color the night predation events (a), and in relation to the time of the day (b). b, Each dot represents a single predation event, dot color indicates hourly median temperature (on the ground, next to the nest) at the time of predation. Lines represent predicted isotherms (based on ground temperatures recorded next to nests). The color scale represents the recorded range of hourly median ground temperatures in the study area, and the gray polygons indicate night (sun >6° below the horizon). Note that no nests were predated when ground temperatures >45℃. c, Increase in probability of night predation across the breeding season. The line with shaded area represents the predicted relationship with 95%CrIs based on the joint posterior distribution of 5,000 simulated values generated by the “sim” function in R (Gelman et al., 2016) from the output of the binomial model (Table A4a). The dots represent single cases of the day (gray) and night (black) predation

**Figure 5:**
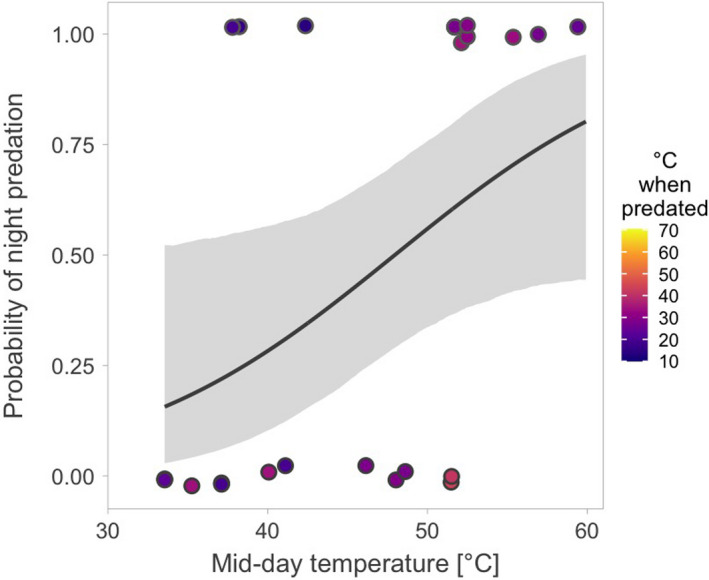
Change in diel timing of predation on red‐wattled lapwing nests in relation to midday temperatures. Each dot represents a single predation event. Top dots represent night predation (sun >6° below the horizon), and bottom dots represent daylight predation. Dot color indicates median hourly temperature (on the ground, next to the nest) at the time of predation, and the color scale represents the range of hourly median ground temperatures recorded within the study area. Note that ground temperatures at the time of predation never exceed 45℃, despite higher ground temperatures during daylight hours being common later in the breeding season (Figures 1 and 3b). The line with shaded area represents the predicted relationship with 95%CrIs based on the joint posterior distribution of 5,000 simulated values generated by the “sim” function in R (Gelman et al., 2016) from the output of the binomial model (Table A4b)

We continuously monitored 230 nests (52% of nests) for an average of 10.3 days (median = 7.7, range: 1.2h – 44 days). During the 2,779 continuously monitored nest‐days, 25 nests were predated. Of the 25 predation events recorded via the continuous monitoring, 12 nests (48%) were predated during the night (the sun was more than 6° below the horizon; Figure 3a). Also, 64% of nests (16 out of 25) were predated during the first part of the day (between midnight and midday; Figure 3a, Table A3a), and nests tended to be predated around sunrise (Figure 3b, Table A3b). Early in the season, nests were predated mainly during the day, while later in the season mainly during the night (Figure 4, Table A4a). Nests were never predated when the ground temperatures exceeded 45℃ (Figure 4b). Accordingly, the probability of night predation increased with increasing midday temperatures (Figure 5, Table A4b). We were unable to statistically distinguish the effect of season and temperature (Table A4c, d), although the model containing only the breeding season seemed the one most supported by the data and twice as likely as the second‐best model with midday temperature (Table A5). The AICc difference between these two models was only 1.48, suggesting that the models were nearly identical.

## 4 DISCUSSION

Our results indicate that the red‐wattled lapwings, breeding in the subtropical, arid, artificial habitat, had a low nest predation rate that was constant over the breeding season. Our data further revealed that the predation events tended to concentrate around sunrise, and night predation was more common later in the season when midday temperatures are high.

### 4.1 Daily nest predation rate

The relatively low nest predation rate (~25%) in our red‐wattled lapwing population is reminiscent of low predation rates found in the suburban population of red‐wattled lapwings breeding on rooftops in India (15%, Sethi et al., 2011), suburban and agricultural population of masked lapwings (*Vanellus miles*) from Australia (0% and 5%, Cardilini et al., 2013), and the population of spur‐winged lapwings (*Vanellus spinosus*) nesting in agricultural landscape and rooftops in Israel (14%, Yogev et al., 1996; 31%, Yogev & Yom‐tov, 1997). Note that most of these predation estimates represent apparent predation rate (not controlled for observation period) and are based on limited sample sizes. Nonetheless, if the reported findings are close to reality, lapwings of genus *Vanellus* may be flexible and well adapted for the suburban and human‐altered habitats. Such adaptations may include colonization of safe sites (such as islands) with generally low density of predators (Sethi et al., 2011; Yogev & Yom‐tov, 1997) and/or an active nest defense (Królikowska et al., 2016; Larsen, 1991).

In contrast, the relatively low nest predation rate in our red‐wattled lapwing population (~25%) dramatically contrasts with the 60% nest predation rate of red‐wattled lapwings breeding in rural India (Sethi et al., 2011), as well as with the nest predation rate of other related subtropical plover species (35%–75%; Makrigianni et al., 2008; Lomas et al., 2014; AlRashidi, 2016; Mishra et al., 2020). The low predation rate also contrasts with nest predation rate of most other plovers and shorebirds (Šálek & Šmilauer, 2002; Watson et al., 2006; Cepáková et al., 2007; Macdonald & Bolton, 2008; Sheldon et al., 2013; Mayfield based predation rates in Vojtěch Kubelka et al., 2018; and Bulla et al., 2019), as well as with nest predation rate of many other birds breeding in arid and also nonarid environments (Freeman et al., 2020; Mezquida & Marone, 2001; Shkedy & Safriel, 1992; Weidinger, 2002).

Given that our red‐wattled lapwing population breeds in human‐altered habitat, it may be debated whether the reported nest predation rate is comparable with the nest predation rate from other populations, species, or geographical regions. There are two reasons why we believe that our results are comparable. First, pristine habitats are becoming increasingly scarce and many shorebird species (e.g., European population of northern lapwings or black‐tailed godwits; Beintema, 1986; Kubelka, Zámečník, et al., 2018) breed in or depend on human‐altered habitats (e.g., arable fields, fishponds, or intensively managed meadows). Red‐wattled lapwings breeding on Arabian Peninsula and other plover species are not an exception (Cardilini et al., 2013; Narwade et al., 2010; Yogev & Yom‐tov, 1997). Second, even seemingly pristine study sites are often located in accessible regions, close to roads and cities (Bulla et al., 2014; Liebezeit et al., 2009; Liebezeit & Zack, 2009) that attract mammalian and avian predators. In other words, human‐altered habitats are currently “natural” breeding habitats for red‐wattled lapwings and for many avian species, and hence, we consider our results representative of the red‐wattled lapwing population and comparable with other nest predation data. If our nest predation rates are comparable with those from other studies, the low predation rate in our subtropical population deviates from the general assumption about latitudinal gradient in predation rates that expects the increase in nest predation rates from the north to the south (Ricklefs, 1969; Stutchbury & Morton, 2013). However, whether our finding holds for other subtropical species, especially those with less active nest‐defense strategies, or is just an exception to the rule requires further investigations. Furthermore, it might be worth investing whether predation rates follow gradients of human‐altered landscapes and whether predation rates differ between landscapes designated to support wildlife and those altered or build by humans for other purposes (e.g., agriculture, recreation).

### 4.2 Diel timing of nest predation

The predation events were distributed evenly across day and night, with a tendency for higher predation around sunrise (Figure 3; Table A3). The lack of distinct day‐night difference is surprising and goes against our initial prediction. First, red‐wattled lapwings actively protect their nests against daylight predators, often in groups of up to seven individuals (Kaur & Khera, 2017; Narwade et al., 2010, own observations). Second, the closely related northern lapwings—breeding in the temperate region and having a two to three times higher nest predation rate (Macdonald & Bolton, 2008; Šálek & Šmilauer, 2002)—also drive away approaching predators during daylight, but ~82% of nest predation events occur at night (Brynychová et al., 2020). It is unclear whether such difference arises because red‐wattled lapwings face a different community of predators (Table A1, versus predators in Brynychová et al., 2020) and/or might be less effective in driving away some predators than northern lapwings. To identify nest predators, we continuously video‐recorded ~116 days of incubation, but did not record a single predation event. Knowing who predates the red‐wattled lapwing nests is essential for clarifying why (despite active nest defense) their nests are predated around the clock.

The lack of overall differences between day and night predation are described, but not formally tested, from the subarctic and arctic regions (Laidlaw et al., 2020; Tulp & Schekkerman, 2001), and anecdotal evidence suggests that the lack of diel pattern in nest predation may be found also in species from regions with a similar composition of predator community (Kosztolanyi et al., 2009; Shkedy & Safriel, 1992). Moreover, around the clock predation is common in ground‐nesting passerines (Pietz et al., 2012; Praus & Weidinger, 2010; Weidinger, 2010), as well as in small shorebirds, that do not actively deter predators (Ekanayake, Weston, et al., 2015; Macdonald & Bolton, 2008).

Despite the overall even distribution of predation events across day and night (i.e., despite fairly constant diel predation pattern; Figure 3), and although daily predation rate changed little over the breeding season (Table A2), the diel timing of predation changed over the breeding season (Figure 4, Table A4 and A5). Specifically, daylight predation nearly disappeared, and the probability of night predation increased, as the season progressed. We offer two (mutually nonexclusive) explanations of this pattern.

**Figure A2:**
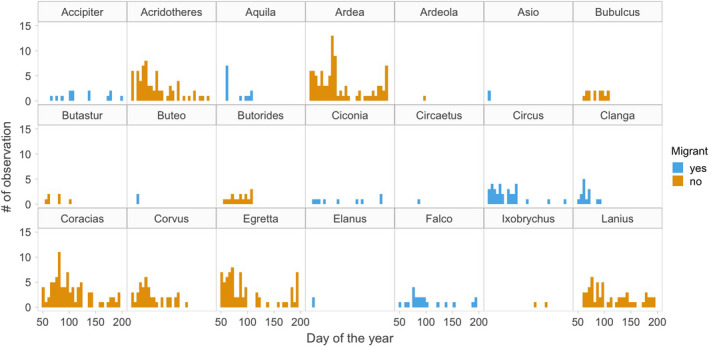
Changes in observations of potential avian predators of red‐wattled lapwing nests over the breeding season. Bars represent a number of observations of a given genus during each day of the breeding season based on all eBird data (*N* = 557 observations) available for Dubai until 1. March 2021, color indicates whether the observed species migrate through or out of the study area during breeding season (blue) or not (orange) (eBird, 2020). Note that despite local (non‐migrating) populations, individuals of the given genus may migrate through the study area. Number of observations are used, instead of the number of seen individuals, because the latter varies between observations and in 74% of observations only one or two individuals were observed, or the number of individuals was unreported. In 5% of observation >10 individuals were observed. Although these data confirm the suspected pattern with migrating species disappearing over the breeding season, the pattern can also reflect increasing mid‐day temperatures accompanied by a decrease in bird watching activities of local inhabitants

First, some birds of prey (daytime predators, such as harriers, kites, and eagles; Table A1, eBird, 2020) migrate from and through the study area early in the breeding season while mammalian predators (mostly nocturnal predators) stay year‐round (Table A1). The lack of migratory birds of prey later in the breeding season certainly reduces predation pressure during the day (Figure A2).

Second, the ambient and surface temperatures dramatically increase over the breeding season (Figure 1) to the point where midday activity of endotherms is close to impossible (Abdu et al., 2018; Albright et al., 2017; Streicher et al., 2017). During such high temperatures, lapwing parents incubate (often rather shade) their eggs continuously to avoid lethal overheating of the embryos (Brown & Downs, 2003; Grant, 1982). Such continuous presence of parents on the nest may protect the nest from smaller avian predators that do not depart the study area—such as common maynas (*Acridotheres tristis*), southern gray shrikes (*Lanius meridionalis*), or Indian rollers (*Coracias benghalensis*)—that can predate eggs, but not the incubating adults (Feare et al., 2015; Verboven et al., 2001). Perhaps more importantly, to minimize energy expenditure and other costs, most animals are inactive during the hottest part of the day (Alagaili et al., 2020; Brown & Downs, 2003; Streicher et al., 2017). Indeed, we found that all predation events with a known time of predation occurred when temperatures were lower than 45℃ (Figure 4b). In other words, when midday temperatures were high, predation was more likely to occur at cooler parts of the day, usually at night (Figures 4, 5; Table A4).

We speculate that the departure of migratory avian predators might be less important in driving the temporal trend in diel timing of predation than the increase in temperatures. First, if migratory birds of prey were the key nest predators, their departure from the study site would decrease daily nest predation across the breeding season (Shkedy & Safriel, 1992), which was not the case (Figure 4a, Table A2). However, the potential decrease of seasonal change in daily nest predation rate might be masked by increase in predation pressure from other predator communities. For example, later in the season when days get extremely hot, resident avian predators (Table A1)—such as egrets and herons (*Ardea* spp.), brown‐necked ravens (*Corvus ruficollis*), common maynas, gray shrikes, or Indian rollers—concentrate around the artificial water bodies and hence in the vicinity of lapwing nests (personal observation). Also, the offspring of these predators disperse (Patnode & White, 1992; Sloan et al., 1998). Second, common nonmigratory predators (that hunt by day) live within the study area (Table A1), which lends support to the idea that temperature extremes are driving the seasonal increase of night nest predation. Third, most migratory avian predators start leaving the study area in March and are gone by the end of April (Figure A2; Table A1, eBird, 2020). However, despite the absence of migratory predators, we recorded cases of daylight predation in May, and daylight predation disappeared only from June onwards (Figure 4b). In contrast, ground temperatures gradually increase over the whole breeding season until the end of July (Figure 1). Importantly, nest predation events start occurring during the colder parts of the day as midday temperatures increase and well before the migratory predators leave the study site (Figure 4b). Thus, midday temperature is likely a stronger driver for the diel change in nest predation than the absence of migratory predators. Nevertheless, whether such change in the timing of predation—linked to midday temperatures—is present in other locally breeding species or avian and nonavian species from other hot environments awaits further investigations.

Regardless of the likely drivers of the changes in the diel pattern of nest predation, the finding generates three predictions (worth future investigations) about the behavior of incubating red‐wattled lapwing parents and parents of any other biparentally incubating species experiencing a similar diel pattern of nest predation. First, given the seasonal changes in the diel pattern of nest predation, we expect seasonal changes in the diel pattern of nest attendance. Specifically, we expect parents to reduce their activity around the nest at night (i.e., increase constancy of incubation, decrease number of nest reliefs), as the season progresses because (i) active nest‐defense during the night is unlikely (e.g., because a parent sees an approaching predator only at a short distance) and (ii) activity at the nest increases nest predation (Martin et al., 2000; Smith et al., 2012). Second, as the season progresses and temperatures increase, we expect the parents to reduce their alertness during the day, spending more time preening and sleeping on the nest, similarly to the northern lapwings (Brynychová et al., 2020). Also, an off‐nest parent on the watch for predators may not be necessary. The off‐nest parent may thus forage, preen, or sleep instead.

## 5 CONCLUSION

We found nest predation to be relatively low in a subtropical population of the poorly studied red‐wattled lapwing, breeding in an arid, artificial habitat. Such low predation rate contrasts with higher predation rates found in many related and unrelated species breeding both in the desert and other habitats (Mezquida & Marone, 2001; Mayfield based predation rates in Bulla et al., 2019; Freeman et al., 2020; Kubelka, Zámečník, et al., 2018). Although we found little variation in daily nest predation rate across the breeding season, the probability of night predation increased over the season, likely due to the extreme heat during the midday. These results help us understand the evolution of different defense behaviors and highlight the need for continuous monitoring to reveal the temporal pattern of predation on multiple time scales, as well as the need for further studies on the timing of predation to evaluate whether the seasonal pattern in temperature‐driven predation is a general rule or an exception to the rule.

## CONFLICT OF INTEREST

We have no competing interests.

## AUTHOR CONTRIBUTIONS

**Martin Sládeček:** Conceptualization (lead); Data curation (lead); Formal analysis (lead); Funding acquisition (equal); Investigation (equal); Methodology (lead); Project administration (equal); Resources (equal); Software (lead); Validation (lead); Visualization (lead); Writing‐original draft (lead); Writing‐review & editing (lead). **Kateřina Brynychová:** Data curation (equal); Funding acquisition (equal); Investigation (equal); Project administration (equal); Resources (equal); Writing‐original draft (supporting). **Esmat Elhassan:** Conceptualization (supporting); Funding acquisition (lead); Investigation (lead); Project administration (equal); Resources (equal); Writing‐original draft (supporting). **Miroslav E Salek:** Conceptualization (equal); Funding acquisition (lead); Investigation (equal); Project administration (lead); Resources (lead); Supervision (lead); Writing‐original draft (equal); Writing‐review & editing (equal). **Veronika Janatová:** Data curation (supporting); Investigation (equal); Project administration (equal); Writing‐original draft (supporting). **Eva Vozabulová:** Funding acquisition (equal); Investigation (equal); Project administration (equal); Resources (equal); Writing‐original draft (supporting). **Petr Chajma:** Data curation (supporting); Investigation (equal); Project administration (supporting); Writing‐original draft (supporting). **Veronika Firlová:** Data curation (supporting); Investigation (equal); Project administration (equal); Writing‐original draft (supporting); Writing‐review & editing (supporting). **Lucie Pešková:** Investigation (equal); Project administration (equal); Writing‐original draft (supporting). **Aisha Almuhery:** Resources (equal); Writing‐review & editing (equal). **Martin Bulla:** Conceptualization (equal); Data curation (equal); Formal analysis (lead); Methodology (equal); Software (lead); Supervision (lead); Validation (lead); Visualization (lead); Writing‐original draft (lead); Writing‐review & editing (lead).

### OPEN RESEARCH BADGES

This article has been awarded Open Materials, Open Data Badges. All materials and data are publicly accessible via the Open Science Framework at https://doi.org/10.17605/OSF.IO/BQ4DR (Sladeček & Bulla, 2021).

## Figures and Tables

**Picture A1:**
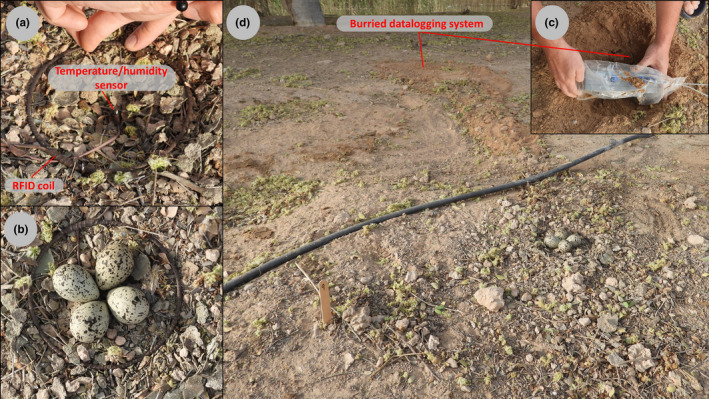
Installation of the ZAYDA incubation monitoring system on a red‐wattled lapwing nest. Minute temperature/humidity probe is placed between the eggs (a), RFID coil around the eggs (a, b), the system, including the cables, is hidden under the ground (c, d)

## Data Availability

All data and computer code to replicate our analyses, as well as plots of model assumptions, are freely available from OSF: https://doi.org/10.17605/OSF.IO/BQ4DR (Sladeček & Bulla, 2021).

## 1

**Table A1:** Potential predators of red‐wattled lapwing eggs in the study site at Al Marmoom Conservation Reserve, Dubai, UAE

Common name	Scientific name	Abundance	Activity	Present
**Montagu's harrier**	***Circus pygargus***	**Regular, but not abundant**	**Day**	**October–March**
**Pallid harrier**	***Circus macrourus***	**Regular, but not abundant**	**Day**	**October–March**
**Black‐eared kite**	***Milvus lineatus***	**Rare**	**Day**	**October–March**
**Western marsh harrier**	***Circus aeruginosus***	**Common and abundant**	**Day**	**September–April**
Common mayna	*Acridotheres tristis*	Common and abundant	Day	Year‐round
Common moorhen	*Gallinula chloropus*	Common and abundant	Day	Year‐round
Brown‐necked raven	*Corvus ruficollis*	Common and abundant	Day	Year‐round
Herons/Egrets	*Ardea spp*.	Common and abundant	Day	Year‐round
Indian roller	*Coracias benghalensis*	Regular, but not abundant	Day	Year‐round
Common kestrel	*Falco tinnunculus*	Regular, but not abundant	Day	Year‐round
Southern grey shrike	*Lanius meridionalis*	Regular, but not abundant	Day	Year‐round
Feral cats	*Felis catus*	Common and abundant	Day/night	Year‐round
Black rat	*Rattus rattus*	Common and abundant	Night	Year‐round
Arabian red fox	*Vulpes v. arabica*	Regular, but not abundant	Night	Year‐round
Desert monitor	*Varanus griseus*	Regular, but not abundant	Day	Year‐round

Note

Avian predators are at the top, other predators at the bottom. The species are ordered according to their presence in the study area and from most to least abundant (based on Dubai municipality internal reports and our haphazard observations). Migratory predators are in bold. Note that none of the migratory predators forages at night.

**Table A2:** Daily predation rate on red‐wattled lapwing nests

					Logit scale						
Model	Effect				Estimate	95% CrI		AICc	∆AICc^a^	*wi* ^b^	ER^c^
(a)	(Intercept)				−4.65	−4.89	−4.42	615.86	0	0.7	1
(b)	(Intercept)				−4.44	−5.19	−3.67	617.54	1.68	0.3	2.33
	Season				0	−0.01	0				

Note

Shown are the posterior estimates (medians) of the effect sizes with the 95% credible intervals (CrI) from a posterior distribution of 5000 simulated values generated by the ”sim” function in R. Response variable indicates whether a nest was predated (0 or 1) and the number of days in which a nest survived as a binomial denominator. The intercept in (a) represent daily predation rate. The AICc comparison was performed with the “AICc” function from the “MuMIn” R‐package (Kamil Bartoń 2020). *N* = 444 nests of red‐wattled lapwings from a population near Dubai.

^a^ The difference in AICc between the first‐ranked model and the given model.

^b^ Akaike weight—the weight of evidence that a given model is the best approximating model (i.e. probability of the model).

^c^ Evidence ratio—the model weight of the first‐ranked model relative to that of the given model (i.e. how many times is the first‐ranked model more likely than the given model).

**Table A3:** Diel pattern of predation on red‐wattled lapwing nests

Model	Response	Error structure	Effect	Estimate	95%CrI	
(a) Time of day	Predation per hour	Poisson	(Intercept)	−0.087	−0.532	0.356
			sin(time)	0.533	−0.035	1.124
			cos(time)	0.481	−0.11	1.072
(a) Time of day	Predation per hour	Gaussian	(Intercept)	1.039	0.469	1.63
			sin(time)	0.535	−0.283	1.335
			cos(time)	0.471	−0.335	1.292
(b) Time relative to sunrise	Predation per hour	Poisson	(Intercept)	0.709	0.043	1.368
			Absolute time from sunrise	−0.121	−0.246	0
(b) Time relative to sunrise	Predation per hour	Gaussian	(Intercept)	1.84	0.848	2.798
			Absolute time from sunrise	−0.13	−0.278	0.014

Note

Shown are the posterior estimates (medians) of the effect sizes with the 95% credible intervals (CrI) from a posterior distribution of 5000 simulated values generated by the ”sim” function in R. Response is specified as a number of predation events per hour of the day (a; *N* = 24 h) and per each hour, centered around sunrise (b; *N* = 23 h).

**Table A4:** Probability of night predation on red‐wattled lapwing nests

Model	Response	Error structure	Effect	Estimate	95%CrI	
(a) Season	Night predation (0,1)	Binomial	(Intercept)	−3.195	−6.058	−0.419
			Season	0.024	0.004	0.045
	Night predation (0,1)	Gaussian	(Intercept)	−0.202	−0.746	0.333
			Season	0.005	0.001	0.009
(b) Midday Temperature	Night predation (0,1)	Binomial	(Intercept)	−5.629	−11.149	−0.331
			Midday temperature	0.117	0.008	0.23
	Night predation (0,1)	Gaussian	(Intercept)	−0.717	−1.823	0.395
			Midday temperature	0.026	0.002	0.049
(c) Midday Temperature & season	Night predation (0,1)	Binomial	(Intercept)	−3.461	−9.5	2.657
			Midday temperature	0.01	−0.19	0.21
			Season	0.023	−0.015	0.06
	Night predation (0,1)	Gaussian	(Intercept)	−0.276	−1.597	1.056
			Midday temperature	0.002	−0.042	0.048
(d) Residual midday temperature & season	Night predation (0,1)	Binomial	Season	0.005	−0.003	0.013
			(Intercept)	−3.229	−5.966	−0.489
			Residual temperature	0.01	−0.19	0.208
			Season	0.025	0.004	0.044
	Night predation (0,1)	Gaussian	(Intercept)	−0.199	−0.743	0.344
			Residual temperature	0.002	−0.042	0.047
			Season	0.005	0.001	0.009
(e) Residual season & midday temperature	Night predation (0,1)	Binomial	(Intercept)	−5.678	−10.952	−0.431
			Residual season	0.023	−0.015	0.06
			Midday temperature	0.118	0.011	0.227
	Night predation (0,1)	Gaussian	(Intercept)	−0.733	−1.766	0.281
			Residual season	0.005	−0.002	0.013
			Midday temperature	0.026	0.005	0.047
(f) Residual temperature	Midday temperature	Gaussian	(Intercept)	26.9	21.61	32.14
			Season	0.16	0.12	0.2
(g) Residual season	Season	Gaussian	(Intercept)	−94.24	−152.61	−38.65
			Midday temperature	4.7	3.53	5.9

Note

Shown are the posterior estimates (medians) of the effect sizes with the 95% credible intervals (CrI) from a posterior distribution of 5000 simulated values generated by the ”sim” function in R. *N* = 25 nests. Note that residuals from (f) were used in (d) and residual from (g) in (e).

**Table A5:** Probability of night predation on red‐wattled lapwing nests—AICc model comparison

Model	Predictors	AICc	∆AICc^a^	*wi* ^b^	ER^c^
(a) Season	Season	32.53	0	0.43	1
(b) Midday Temperature	Midday temperature	34.01	1.48	0.21	2.05
(c) Midday T & season	Season & Midday temperature	35.12	2.59	0.12	3.58
(d) Residual T & season	Season & Residual midday temperature	35.12	2.59	0.12	3.58
(e) Residual season & T	Midday temperature & Residual season	35.12	2.59	0.12	3.58

Note

Model names correspond to the model names in Table A4. The AICc comparison was performed with the AICc function from the MuMIn R‐package (Kamil Bartoń 2020) and separately for binomial and Gaussian models, which generated identical results.

^a^ The difference in AICc between the first‐ranked model and the given model.

^b^ Akaike weight—the weight of evidence that a given model is the best approximating model (i.e. probability of the model).

^c^ Evidence ratio—the model weight of the first‐ranked model relative to that of the given model (i.e. how many times is the first‐ranked model more likely than the given model).

## References

[ece38025-bib-0001] Abdu, S. , McKechnie, A. E. , Lee, A. T. K. , & Cunningham, S. J. (2018). Can providing shade at water points help Kalahari birds beat the heat? Journal of Arid Environments, 152(1), 21–27. 10.1016/j.jaridenv.2018.01.018

[ece38025-bib-0002] Aebischer, N. J. (1999). Multi‐way comparisons and generalized linear models of nest success: Extensions of the mayfield method. Bird Study, 46, S22–S31. 10.1080/00063659909477228

[ece38025-bib-0003] Alagaili, A. N. , Bennett, N. C. , Amor, N. M. , & Hart, D. W. (2020). The locomotory activity patterns of the arid‐dwelling desert hedgehog, *Paraechinus aethiopicus*, from Saudi Arabia. Journal of Arid Environments, 177, 104141. 10.1016/j.jaridenv.2020.104141

[ece38025-bib-0004] Albright, T. P. , Mutiibwa, D. , Gerson, A. R. , Smith, E. K. , Talbot, W. A. , O’Neill, J. J. , McKechnie, A. E. , & Wolf, B. O. (2017). Mapping evaporative water loss in desert passerines reveals an expanding threat of lethal dehydration. Proceedings of the National Academy of Sciences, 114(9), 2283–2288. 10.1073/pnas.1613625114 PMC5338552 28193891

[ece38025-bib-0005] AlRashidi, M. (2016). Breeding biology of the Kentish Plover *Charadrius alexandrinus* in the Sabkhat Al‐Fasl Lagoons, Saudi Arabia (Aves: Charadriiformes). Zoology in the Middle East, 62(2), 105–111. 10.1080/09397140.2016.1182771

[ece38025-bib-0006] Anderson, D. R. (2008). Model based inference in the life sciences: A primer on evidence. Springer Science+Business Media.

[ece38025-bib-0007] Bakner, N. W. , Schofield, L. R. , Cedotal, C. , Chamberlain, M. J. , & Collier, B. A. (2019). Incubation recess behaviors influence nest survival of Wild Turkeys. Ecology and Evolution, 9(24), 14053–14065. 10.1002/ece3.5843 31938503 PMC6953688

[ece38025-bib-0008] Bartoń, K. (2019). MuMIn: Multi‐Model Inference R package. Available at: http://mumin.r‐forge.r‐project.org/

[ece38025-bib-0009] Batáry, P. , Winkler, H. , & Báldi, A. (2004). Experiments with artificial nests on predation in reed habitats. Journal of Ornithology, 145(1), 59–63. 10.1007/s10336-003-0010-9

[ece38025-bib-0010] Beintema, J. A. (1986). Man‐Made Polders in the Netherlands: A Traditional Habitat for Shorebirds. Colonal Waterbirds, 9(2), 196–202. 10.2307/1521213

[ece38025-bib-0011] Bivand, R. , & Lewin‐Koh, N. (2020). maptools: Tools for handling spatial objects. R package. Available at: http://maptools.r‐forge.r‐project.org/

[ece38025-bib-0012] Brown, M. , & Downs, C. T. (2003). The role of shading behaviour in the thermoregulation of breeding crowned plovers (*Vanellus coronatus*). Journal of Thermal Biology, 28(1), 51–58. 10.1016/S0306-4565(02)00036-0

[ece38025-bib-0013] Brown, S. C. , Gates, H. R. , Liebezeit, J. R. , Smith, P. A. , Hill, B. L. , & Lanctot, R. B. (2014). Arctic Shorebird Demographics Network Breeding Camp Protocol, Unpubl. paper by U.S. Fish and Wildlife Service and Manomet Center for Conservation Sciences, p. 118.

[ece38025-bib-0014] Brynychová, K. , Šálek, M. E. , Vozabulová, E. , & Sládeček, M. (2020). Daily rhythms of female self‐maintenance correlate with predation risk and male nest attendance in a biparental wader. Journal of Biological Rhythms, 35(5), 489–500. 10.1177/0748730420940465 32677476

[ece38025-bib-0015] Bulla, M. , Reneerkens, J. , Weiser, E. L. , Lanctot, R. B. , & Kempenaers, B. (2019). Supporting information for comment on “Global pattern of nest predation is disrupted by climate change in shorebirds, Open Science Framework. Available at: https://osf.io/x8fs6/ 10.1126/science.aaw8529 31196986

[ece38025-bib-0016] Bulla, M. , Valcu, M. , Dokter, A. M. , Dondua, A. G. , Kosztolányi, A. , Rutten, A. L. , Helm, B. , Sandercock, B. K. , Casler, B. , Ens, B. J. , Spiegel, C. S. , Hassell, C. J. , Küpper, C. , Minton, C. , Burgas, D. , Lank, D. B. , Payer, D. C. , Loktionov, E. Y. , Nol, E. , … Kempenaers, B. (2016). Unexpected diversity in socially synchronized rhythms of shorebirds. Nature, 540(7631), 109–113. 10.1038/nature20563 27880762

[ece38025-bib-0017] Bulla, M. , Valcu, M. , Rutten, A. L. , & Kempenaers, B. (2014). Biparental incubation patterns in a high‐Arctic breeding shorebird: How do pairs divide their duties? Behavioral Ecology, 25(1), 152–164. 10.1093/beheco/art098 24347997 PMC3860833

[ece38025-bib-0018] Cardilini, A. P. A. , Weston, M. A. , Nimmo, D. G. , Dann, P. , & Sherman, C. D. H. (2013). Surviving in sprawling suburbs: Suburban environments represent high quality breeding habitat for a widespread shorebird. Landscape and Urban Planning, 115, 72–80. 10.1016/j.landurbplan.2013.04.001

[ece38025-bib-0019] Caro, T. (2005). Antipredator defences in birds and mammals. University of Chicago Press.

[ece38025-bib-0020] Cepáková, E. , Šálek, M. , Cepák, J. , & Albrecht, T. (2007). Breeding of Little Ringed Plovers *Charadrius dubius* in farmland: Do nests in fields suffer from predation? Bird Study, 54(2), 284–288. 10.1080/00063650709461487

[ece38025-bib-0021] Cervencl, A. , Esser, W. , Maier, M. , Oberdiek, N. , Thyen, S. , Wellbrock, A. , & Exo, K.‐M. (2011). Can differences in incubation patterns of Common Redshanks *Tringa totanus* be explained by variations in predation risk? Journal of Ornithology, 152(4), 1033–1043. 10.1007/s10336-011-0696-z

[ece38025-bib-0022] DeGregorio, B. A. , Sperry, J. H. , Ward, M. P. , & Weatherhead, P. J. (2015). Wait until dark? Daily activity patterns and nest predation by snakes. Ethology, 121(12), 1225–1234. 10.1111/eth.12435

[ece38025-bib-0023] eBird . (2020). eBird: An online database of bird distribution and abundance [web application]. Available at: www.ebird.org [Accessed 20 November 2020]

[ece38025-bib-0024] Eggers, S. , Griesser, M. , & Ekman, J. (2008). Predator‐induced reductions in nest visitation rates are modified by forest cover and food availability. Behavioral Ecology, 19(5), 1056–1062. 10.1093/beheco/arn063

[ece38025-bib-0025] Ekanayake, K. B. , Weston, M. A. , Nimmo, D. G. , Maguire, G. S. , Endler, J. A. , & Küpper, C. (2015). The bright incubate at night: Sexual dichromatism and adaptive incubation division in an open‐nesting shorebird. Proceedings of the Royal Society B: Biological Sciences, 282(1806), 20143026. 10.1098/rspb.2014.3026 PMC4426615 25854884

[ece38025-bib-0026] Ekanayake, K. B. , Whisson, D. A. , Tan, L. X. L. , & Weston, M. A. (2015). Intense predation of non‐colonial, ground‐nesting bird eggs by corvid and mammalian predators. Wildlife Research, 42(6), 518–528. 10.1071/WR15080

[ece38025-bib-0027] Feare, C. J. , Lebarbenchonb, C. , Dietrichc, M. , & Larosed, C. S. (2015). Predation of seabird eggs by Common Mynas *Acridotheres tristis* on Bird Island, Seychelles, and its broader implications. Bulletin of the African Bird Club, 22(2), 162–170.

[ece38025-bib-0028] Freeman, B. G. , Scholer, M. N. , Boehm, M. M. A. , Heavyside, J. , & Schluter, D. (2020). Adaptation and latitudinal gradients in species interactions: Nest predation in birds. American Naturalist, 196(6), E160–E166. 10.1086/711415 33211562

[ece38025-bib-0029] Gelman, A. , & Hill, J. (2007). Data analysis using regression and multilevel/hierarchical models. Cambridge University Press. 10.2277/0521867061

[ece38025-bib-0030] Gelman, A. , Yu‐Sung, S. U. , Yajima, M. , Hill, J. , Pittau, M. G. , Kerman, J. , Zheng, T. , & Dorie, V. (2016). Data Analysis Using Regression and Multilevel/Hierarchical Models. CRAN Repository, 1–53. Available at: https://cran.r‐project.org/package=arm

[ece38025-bib-0031] Grant, G. S. (1982). Avian incubation: Egg temperature, nest humidity, and behavioral thermoregulation in a hot environment. Ornithological Monographs, 30(30), 1–82. 10.2307/40166669

[ece38025-bib-0032] Javůrková, V. , Hořák, D. , Kreisinger, J. , Klvaňa, P. , & Albrecht, T. (2011). Factors affecting sleep/vigilance behaviour in incubating mallards. Ethology, 117(4), 345–355. 10.1111/j.1439-0310.2011.01878.x

[ece38025-bib-0033] Kämmerle, J.‐L. , Rondeaux, S. , & Storch, I. (2020). Circadian activity patterns of red foxes (*Vulpes vulpes*) in montane forests under different culling regimes. Mammal Research, 65(3), 615–619. 10.1007/s13364-020-00496-w

[ece38025-bib-0034] Kaur, M. , & Khera, K. (2017). On the fundamentals of breeding biology and present threats to red wattled lapwing (*Vanellus indicus*) in agricultural landscape of Punjab. Journal of Entomology and Zoology Studies, 5(4), 1501–1506.

[ece38025-bib-0035] Kentie, R. , Both, C. , Hooijmeijer, J.C. & Piersma, T. (2015). Management of modern agricultural landscapes increases nest predation rates in Black‐tailed Godwits Limosa limosa. Ibis, 157(3), 614–625. 10.1111/ibi.12273

[ece38025-bib-0036] Knief, U. , & Forstmeier, W. (2018). Violating the normality assumption may be the lesser of two evils. bioRxiv. 10.1101/498931 PMC8613103 33963496

[ece38025-bib-0037] Kosztolanyi, A. , Javed, S. , Küpper, C. , Cuthill, I. C. , Al Shamsi, A. , & Székely, T. (2009). Breeding ecology of Kentish Plover *Charadrius alexandrinus* in an extremely hot environment. Bird Study, 56(2), 244–252. 10.1080/00063650902792106

[ece38025-bib-0038] Królikowska, N. , Szymkowiak, J. , & Laidlaw, R. A. (2016). Threat‐sensitive anti‐predator defence in precocial wader, the northern lapwing *Vanellus vanellus* . Acta Ethologica, 19(3), 163–171. 10.1007/s10211-016-0236-1 27738383 PMC5039224

[ece38025-bib-0039] Kubelka, V. , Šálek, M. , Tomkovich, P. , Végvári, Z. , Freckleton, R. P. , & Székely, T. (2018). Data and R codes from: Global pattern of nest predation is disrupted by climate change in shorebirds Dryad. Dryad. 10.5061/dryad.45g90h4 30409881

[ece38025-bib-0040] Kubelka, V. , Zámečník, V. , Slabeyová, K. , Škorpíková, V. , & Šálek, M. (2018). Threats and conservation of meadow‐breeding shorebirds in the Czech Republic and Slovakia. Wader Study, 125(3), 164–174. 10.18194/WS.00124

[ece38025-bib-0041] Laidlaw, R. A. , Gunnarsson, T. G. , Méndez, V. , Carneiro, C. , Þórisson, B. , Wentworth, A. , Gill, J. A. , & Alves, J. A. (2020). Vegetation structure influences predation rates of early nests in subarctic breeding waders. Ibis, 162(4), 1225–1236. 10.1111/ibi.12827

[ece38025-bib-0042] Larsen, T. (1991). Anti‐predator behaviour and mating systems in waders: Aggressive nest defence selects for monogamy. Animal Behaviour, 41(6), 1057–1062. 10.1016/S0003-3472(05)80643-8

[ece38025-bib-0043] Liebezeit, J. R. , Kendall, S. J. , Brown, S. , Johnson, C. B. , Martin, P. , McDonald, T. L. , Payer, D. C. , Rea, C. L. , Streever, B. , Wildman, A. M. , & Zack, S. (2009). Influence of human development and predators on nest survival of tundra birds, Arctic Coastal Plain, Alaska. Ecological Applications, 19(6), 1628–1644. 10.1890/08-1661.1 19769108

[ece38025-bib-0044] Liebezeit, J. , & Zack, S. (2009). Nesting success and nest predators of tundra‐nesting birds in the Prudhoe Bay Oilfield – Long‐term monitoring 2009 report, (December), p. 34.

[ece38025-bib-0045] Lomas, S. C. , Whisson, D. A. , Maguire, G. S. , Tan, L. X. , Guay, P. J. , & Weston, M. A. (2014). The influence of cover on nesting red‐capped plovers: A trade‐off between thermoregulation and predation risk? Victorian Naturalist, 131(4), 115–127.

[ece38025-bib-0046] Mabee, T. J. , Wildman, A. M. , & Johnson, C. B. (2006). Using egg flotation and eggshell evidence to determine age and fate of Arctic shorebird nests. Journal of Field Ornithology, 77(2), 163–172. 10.1111/j.1557-9263.2006.00037.x

[ece38025-bib-0047] Macdonald, M. A. , & Bolton, M. (2008). Predation on wader nests in Europe. Ibis, 150(SUPPL.1), 54–73. 10.1111/j.1474-919X.2008.00869.x

[ece38025-bib-0048] Makrigianni, E. , Sgardelis, S. , Poirazidis, K. , & Athanasiadis, A. (2008). Breeding biology and nesting site selection by the spur‐winged plover *Hoplopterus spinosus* in the Evros Delta, NE Greece. Journal of Natural History, 42(5–8), 333–344. 10.1080/00222930701835225

[ece38025-bib-0049] Martin, T. E. , Scott, J. , & Menge, C. (2000). Nest predation increases with parental activity: Separating nest site and parental activity effects. Proceedings of the Royal Society B: Biological Sciences, 267(1459), 2287–2293. 10.1098/rspb.2000.1281 PMC1690815 11413645

[ece38025-bib-0050] Massaro, M. , Starling‐Windhof, A. , Briskie, J. V. , & Martin, T. E. (2008). Introduced mammalian predators induce behavioural changes in parental care in an endemic New Zealand bird. PLoS One, 3(6), e2331. 10.1371/journal.pone.0002331 18523640 PMC2396284

[ece38025-bib-0051] Mayfield, H. (1961). Nesting success calculated from exposure. The Wilson Bulletin, 73(3), 255–261.

[ece38025-bib-0052] McGuire, R. L. , Lanctot, R. B. , Saalfeld, S. T. , Ruthrauff, D. R. , & Liebezeit, J. R. (2020). Shorebird reproductive response to exceptionally early and late springs varies across sites in arctic alaska. Frontiers in Ecology and Evolution, 8, 1–23. 10.3389/fevo.2020.577652

[ece38025-bib-0053] Meyer, N. , Bollache, L. , Dechaume‐Moncharmont, F.‐X. , Moreau, J. , Afonso, E. , Angerbjörn, A. , Bêty, J. , Ehrich, D. , Gilg, V. , Giroux, M.‐A. , Hansen, J. , Lanctot, R. B. , Lang, J. , Lecomte, N. , McKinnon, L. , Reneerkens, J. , Saalfeld, S. T. , Sabard, B. , Schmidt, N. M. , … Gilg, O. (2020). Nest attentiveness drives nest predation in arctic sandpipers. Oikos, 129(10), 1481–1492. 10.1111/oik.07311

[ece38025-bib-0054] Mezquida, E. T. , & Marone, L. (2001). Factors affecting nesting success of a bird assembly in the central Monte Desert, Argentina. Journal of Avian Biology, 32(4), 287–296. 10.1111/j.0908-8857.2001.320401.x

[ece38025-bib-0055] Mishra, H. , Kumar, V. , & Kumar, A. (2020). Factors influencing nesting success of the river lapwing, *Vanellus duvaucelii* (Lesson, 1826). Avian Biology Research, 13(3), 35–43. 10.1177/1758155920921072

[ece38025-bib-0056] Morton, E. S. (1971). Nest predation affecting the breeding season of the Clay‐colored Robin, a tropical song bird. Science, 171(3974), 920–921. 10.1126/science.171.3974.920 5100205

[ece38025-bib-0057] Narwade, S. , Fartade, M. , & Fartade, K. (2010). Effect of agricultural activities on breeding success of red‐wattled lapwing Vanellus indicus. National Journal of Life Sciences, 7(1), 31–34.

[ece38025-bib-0058] Patnode, K. A. , & White, D. H. (1992). Effects of habitat on avian productivity in abandoned pecan orchards in Southern Georgia. Journal of Field Ornithology, 63(1), 77–85.

[ece38025-bib-0059] Pietz, P. J. , Granfors, D. A. , & Ribic, C. A. (2012). Knowledge gained from video‐monitoring grassland passerine nests. *USGS Northern Prairie Wildlife Research Center*, 254.

[ece38025-bib-0060] Praus, L. , & Weidinger, K. (2010). Predators and nest success of Sky Larks *Alauda arvensis* in large arable fields in the Czech Republic. Bird Study, 57(4), 525–530. 10.1080/00063657.2010.506208

[ece38025-bib-0061] R‐Core‐Team . (2019). R: A Language and Environment for Statistical Computing. R Foundation for Statistical Computing. Retrieved from: http://www.r‐project.org/

[ece38025-bib-0062] Ricklefs, R. E. (1969). An analysis of nesting mortality. Smithsonian Institution Press, Contributions to Zoology, 9(9), 1–48.

[ece38025-bib-0063] Robinson, W. D. , Rompré, G. , & Robinson, T. R. (2005). Videography of Panama bird nests shows snakes are principal predators. Ornitologia Neotropical, 16(2), 187–195.

[ece38025-bib-0064] Šálek, M. , & Šmilauer, P. (2002). Predation on Northern Lapwing *Vanellus vanellus* nests: The effect of populaton density and spatial distribution of nests. Ardea, 90(1), 51–60.

[ece38025-bib-0065] Schielzeth, H. , Dingemanse, N. J. , Nakagawa, S. , Westneat, D. F. , Allegue, H. , Teplitsky, C. , Réale, D. , Dochtermann, N. A. , Garamszegi, L. Z. , & Araya‐Ajoy, Y. G. (2020). Robustness of linear mixed‐effects models to violations of distributional assumptions. Methods in Ecology and Evolution, 11(9), 1141–1152. 10.1111/2041-210X.13434

[ece38025-bib-0066] Sethi, V. K. , Bhatt, D. , Kumar, A. , & Naithani, A. B. (2011). The hatching success of ground‐ and roof‐nesting Red‐wattled Lapwing *Vanellus indicus* in Haridwar, India. Forktail, 27, 7–10.

[ece38025-bib-0067] Sheldon, R. D. , Kamp, J. , Koshkin, M. A. , Urazaliev, R. S. , Iskakov, T. K. , Field, R. H. , Salemgareev, A. R. , Khrokov, V. V. , Zhuly, V. A. , Sklyarenko, S. L. , & Donald, P. F. (2013). Breeding ecology of the globally threatened Sociable Lapwing *Vanellus gregarius* and the demographic drivers of recent declines. Journal of Ornithology, 154(2), 501–516. 10.1007/s10336-012-0921-4

[ece38025-bib-0068] Shkedy, Y. , & Safriel, U. N. (1992). Nest predation and nestling growth rate of two lark species in the Negev Desert, Israel. Ibis, 134(3), 268–272. 10.1111/j.1474-919X.1992.tb03809.x

[ece38025-bib-0069] Sieving, K. E. (2019). Nest Predation and Differential Insular Extinction among Selected Forest Birds of Central Panama. Ecology, 73(6), 2310–2328. 10.2307/1941477

[ece38025-bib-0070] Skórka, P. , Wójcik, J. D. , Martyka, R. , & Lenda, M. (2012). Numerical and behavioural response of Black‐headed Gull *Chroicocephalus ridibundus* on population growth of the expansive Caspian Gull *Larus cachinnans* . Journal of Ornithology, 153(3), 947–961. 10.1007/s10336-012-0824-4

[ece38025-bib-0071] Skutch, A. F. (1985). Clutch size, nesting success, and predation on nests of neotropical birds, reviewed. Ornithological Monographs, 36, 575–594. 10.2307/40168306

[ece38025-bib-0072] Sladeček, M. , & Bulla, M. (2021). Supporting information for 'Diel timing of nest predation changes across breeding season in a subtropical shorebird'. Open Science Framework, 10.17605/OSF.IO/BQ4DR PMC8495801 34646455

[ece38025-bib-0073] Sládeček, M. , Vozabulová, E. , Brynychová, K. , & Šálek, M. E. (2019). Parental incubation exchange in a territorial bird species involves sex‐specific signalling. Frontiers in Zoology, 16(7), 1–12. 10.1186/s12983-019-0306-0 30949226 PMC6431054

[ece38025-bib-0074] Sládeček, M. , Vozabulová, E. , Šálek, M. , & Bulla, M. (2019). Diversity of incubation rhythms in a facultatively uniparental shorebird – the Northern Lapwing. Scientific Reports, 9(4706), 324426. 10.1038/s41598-019-41223-z PMC6423287 30886196

[ece38025-bib-0075] Sloan, S. S. , Holmes, R. T. , & Sherry, T. W. (1998). Depredation rates and predators at artificial bird nests in an unfragmented northern hardwoods forest. The Journal of Wildlife Management, 62(2), 529. 10.2307/3802326

[ece38025-bib-0076] Smith, P. A. , Tulp, I. , Schekkerman, H. , Gilchrist, H. G. , & Forbes, M. R. (2012). Shorebird incubation behaviour and its influence on the risk of nest predation. Animal Behaviour, 84(4), 835–842. 10.1016/j.anbehav.2012.07.004

[ece38025-bib-0077] Sperry, J. H. , Peak, R. G. , Cimprich, D. A. , & Weatherhead, P. J. (2008). Snake activity affects seasonal variation in nest predation risk for birds. Journal of Avian Biology, 39(4), 379–383. 10.1111/j.2008.0908-8857.04451.x

[ece38025-bib-0078] Streicher, S. , Lutermann, H. , Bennett, N. C. , Bertelsen, M. F. , Mohammed, O. B. , Manger, P. R. , Scantlebury, M. , Ismael, K. , & Alagaili, A. N. (2017). Living on the edge: Daily, seasonal and annual body temperature patterns of Arabian oryx in Saudi Arabia. PLoS One, 12(8), 1–15. 10.1371/journal.pone.0180269 PMC5576856 28854247

[ece38025-bib-0079] Stutchbury, B. J. , & Morton, E. S. (2013). Behavioral ecology of tropical birds. Academic Press.

[ece38025-bib-0080] Symes, A. , Taylor, J. , Mallon, D. , Porter, R. , Simms, C. , & Budd, K. (2017). The conservation status and distribution of the breeding birds of the Arabian peninsula, The conservation status and distribution of the breeding birds of the Arabian peninsula, 10.2305/iucn.ch.2015.mra.5.en

[ece38025-bib-0081] Tahajjul Taufique, S. K. , Jha, N. A. , & Kumar, V. (2016). Circadian rhythm determines the timing of activity, and ingestive and grooming behaviours in Indian house crows, *Corvus splendens* . Current Science, 110(5), 897–901. 10.18520/cs/v110/i5/897-901

[ece38025-bib-0082] Tulp, I. , & Schekkerman, H. (2001). Studies on breeding shorebirds at Medusa Bay, Taimyr, in summer 2001. Green World Research, 1–110.

[ece38025-bib-0083] Unzeta, M. , Martin, T. E. , & Sol, D. (2020). Daily nest predation rates decrease with body size in passerine birds. American Naturalist, 196(6), 743–754. 10.1086/711413 33211569

[ece38025-bib-0084] van Paassen, A. G. , Veldman, D. H. , & Beintema, A. J. (1984). A simple device for determination of incubation stages in eggs. Wildfowl, 35(1950), 173–178.

[ece38025-bib-0085] Verboven, N. , Ens, B. J. , & Dechesne, S. (2001). Effect of investigator disturbance on nest attendance and egg predation in Eurasian Oystercatchers. The Auk, 118(2), 503–508. 10.2307/4089811

[ece38025-bib-0086] Visco, D. M. , & Sherry, T. W. (2015). Increased abundance, but reduced nest predation in the chestnut‐backed antbird in Costa Rican rainforest fragments: Surprising impacts of a pervasive snake species. Biological Conservation, 188, 22–31. 10.1016/j.biocon.2015.01.015

[ece38025-bib-0087] Watson, M. et al (2006). Nest survival and productivity of the critically endangered Sociable Lapwing *Vanellus gregarius* . Ibis, 148(3), 489–502. 10.1111/j.1474-919X.2006.00555.x

[ece38025-bib-0088] Weatherhead, P. J. , & Blouin‐demers, G. (2004). Understanding Avian Nest Predation : Why Ornithologists Should Study Snakes. Journal of Avian Biology, 35(3), 185–190.

[ece38025-bib-0089] Weidinger, K. (2002). Interactive effects of concealment, parental behaviour and predators on the survival of open passerine nests. Journal of Animal Ecology, 71(3), 424–437. 10.1046/j.1365-2656.2002.00611.x

[ece38025-bib-0090] Weidinger, K. (2006). Validating the use of temperature data loggers to measure survival of songbird nests. Journal of Field Ornithology, 77(4), 357–364. 10.1111/j.1557-9263.2006.00063.x

[ece38025-bib-0091] Weidinger, K. (2010). Foraging behaviour of nest predators at open‐cup nests of woodland passerines. Journal of Ornithology, 151(3), 729–735. 10.1007/s10336-010-0512-1

[ece38025-bib-0092] Weiser, E. L. , Brown, S. C. , Lanctot, R. B. , Gates, H. R. , Abraham, K. F. , Bentzen, R. L. , Bêty, J. , Boldenow, M. L. , Brook, R. W. , Donnelly, T. F. , English, W. B. , Flemming, S. A. , Franks, S. E. , Gilchrist, H. G. , Giroux, M.‐A. , Johnson, A. , Kendall, S. , Kennedy, L. V. , Koloski, L. , … Sandercock, B. K. (2018). Effects of environmental conditions on reproductive effort and nest success of Arctic‐breeding shorebirds. Ibis, 160(3), 608–623. 10.1111/ibi.12571

[ece38025-bib-0093] Weiser, E. L. , Lanctot, R. B. , Brown, S. C. , Alves, J. A. , Battley, P. F. , Bentzen, R. , Bêty, J. , Bishop, M. A. , Boldenow, M. , Bollache, L. , Casler, B. , Christie, M. , Coleman, J. T. , Conklin, J. R. , English, W. B. , Gates, H. R. , Gilg, O. , Giroux, M.‐A. , Gosbell, K. , … Sandercock, B. K. (2016). “Effects of geolocators on hatching success, return rates, breeding movements, and change in body mass in 16 species of Arctic‐breeding shorebirds. Movement Ecology, 4(1), 10.1186/s40462-016-0077-6 PMC4850671 27134752

[ece38025-bib-0094] Wickham, H. (2016). ggplot2: Elegant graphics for data analysis. Springer‐Verlag.

[ece38025-bib-0095] Wiersma, P. (2020). Red‐wattled Lapwing (*Vanellus indicus*). In J. A. E. del Hoyo et al (eds) Birds of the World. 1.0. Cornell Lab of Ornithology.

[ece38025-bib-0096] Yogev, A. , Ar, A. , & Yom‐tov, Y. (1996). Determination of clutch size and the breeding biology of the spur‐winged plover (*Vanellus spinosus*) in Israel. The Auk, 113(1), 68–73. 10.2307/4088936

[ece38025-bib-0097] Yogev, A. , & Yom‐tov, Y. (1997). Roof Laying By the Spur‐Winged Plover, *Vanellus Spinosus* . Israel Journal of Zoology, 43(1), 87–88. 10.1080/00212210.1997.10688893

